# Family Resilience and Mental and Physical Health Sequelae of Pediatric TBI in Youths

**DOI:** 10.1001/jamanetworkopen.2026.9222

**Published:** 2026-04-13

**Authors:** Zhengyang Zhou, Lindsay Sullivan, Ruoyuan Qian, Bo Lu, Eric A. Sribnick, Gary A. Smith, K. Luan Phan, Scott A. Langenecker, Frederick P. Rivara, Henry Xiang

**Affiliations:** 1 Center for Injury Research and Policy, Abigail Wexner Research Institute, Nationwide Children’s Hospital, Columbus, Ohio; 2 School of Health and Rehabilitation Sciences, The Ohio State University, Columbus; 3 Division of Biostatistics, College of Public Health, The Ohio State University, Columbus; 4 University of Washington, Seattle; 5 College of Medicine, The Ohio State University, Columbus

## Abstract

**Importance:**

Traumatic brain injury (TBI) in children and adolescents is associated with mental and physical health sequelae, including elevated risks of anxiety, depression, persistent headaches, and chronic physical pain. However, factors that may be associated with the risk of these adverse outcomes and with improved recovery remain underexplored.

**Objective:**

To examine associations between medically diagnosed TBI and mental (anxiety and depression) and physical (frequent headaches and chronic pain) health outcomes among US children and adolescents aged 6 to 17 years and to evaluate whether these associations vary by level of family resilience.

**Design, Setting, and Participants:**

This cross-sectional study used data from the 2022 and 2023 National Survey of Children’s Health (NSCH), a nationally representative survey conducted by the US Census Bureau and sponsored by the Health Resources and Services Administration Maternal and Child Health Bureau. The NSCH collects caregiver-reported data on child and adolescent health, family context, and social determinants across all 50 US states and the District of Columbia. This study included children and adolescents aged 6 to 17 years whose parents reported medically diagnosed TBI and a comparison group without medically diagnosed TBI. Children and adolescents with comorbidities were excluded. Survey weights accounted for complex sampling to generate nationally representative estimates. Data analysis was done from June 1 to November 13, 2025.

**Exposures:**

Medically diagnosed TBI.

**Main Outcomes and Measures:**

Current status and severity of medically diagnosed anxiety, depression, frequent or severe headaches (including migraine headaches), and chronic physical pain. Moderators included adverse childhood experiences, child and adolescent flourishing, and family resilience.

**Results:**

Among 33 572 participants, 1195 individuals (3.5%; 47.0% [95% CI, 41.2%-52.9%] female; 46.5% [95% CI, 41.0%-52.0%] aged 15-17 years) had a history of medically diagnosed TBI and 32 377 individuals (96.4%; 54.0% [95% CI, 52.9%-55.1%] female; 51.7% [95% CI, 50.6%-52.8%] aged 6-11 years) had no TBI. Children and adolescents with TBI had a significantly higher prevalence of poor health than those without TBI (eg, current anxiety: 12.9% [95% CI, 9.3%-16.5%] vs 4.7% [95% CI, 4.3%-5.0%]; adjusted prevalence ratio, 1.83 [95% CI, 1.38-2.43]). They had increased odds of current anxiety (adjusted odds ratio [aOR], 1.87 [95% CI, 1.31-2.67]), depression (aOR, 1.98 [95% CI, 1.13-3.47]), frequent headaches (aOR, 7.76 [95% CI, 2.71-22.20]), and chronic pain (aOR, 3.99 [95% CI, 1.81-8.77]). Interaction analyses showed evidence of effect modification on the multiplicative (OR) scale for depression, with higher odds observed at moderate (aOR, 5.64 [95% CI, 1.13-28.20]; *P* = .04) and low (aOR, 6.41 [95% CI, 1.24-33.20]; *P* = .03) resilience levels compared with the reference group (no TBI × high resilience).

**Conclusions and Relevance:**

In this study, family resilience was associated with variation in the association between TBI and depression. These findings support further investigation of family-focused rehabilitation approaches in prospective studies.

## Key Points

**Question:**

Is the association between pediatric traumatic brain injury (TBI) and adverse mental and physical health outcomes modified by family resilience?

**Findings:**

In this cross-sectional study of 33 572 US children and adolescents (aged 6-17 years), those with medically diagnosed TBI had significantly increased odds of anxiety, depression, headaches, and chronic pain. Greater family resilience and child flourishing were associated with decreased odds of these outcomes, particularly depression, whereas adverse childhood experiences were associated with increased odds.

**Meaning:**

In this study, family resilience was associated with decreased odds of depression among children with TBI.

## Teaser

This cross-sectional study examines the association of pediatric traumatic brain injury with adverse mental and physical health outcomes among youths ages 6 to 17 years.

## Introduction

Traumatic brain injury (TBI) in childhood and adolescence is increasingly recognized as a major determinant of long-term mental and physical health, including anxiety, depression, persistent headaches, and chronic pain.^1,2,3,4,5,6^ Prior research suggests that higher rates of anxiety and depression after pediatric TBI may result from disruption of frontal-subcortical and limbic circuits involved in emotional regulation.^6^ Additionally, executive dysfunction and impaired emotional processing after TBI may be associated with further increased risk of persistent internalizing symptoms.^2^ TBI can be associated with sleep disturbances, motor coordination deficits, and sensory impairments that affect daily functioning, academic achievement, and overall quality of life.^1,7^ Despite these risks, few large, population-based studies have comprehensively examined the co-occurrence of mental and physical health sequelae of pediatric TBI or evaluated whether family factors are associated with decreased odds of these adverse outcomes.^8,9,10^ Most prior research has conceptualized TBI primarily as a mechanistic risk factor associated with negative outcomes rather than investigating modifiable psychosocial assets, such as family resilience, that may buffer or exacerbate these associations.

Adverse childhood experiences (ACEs) are well-established risk factors associated with poor mental and physical health across development.^11,12^ In contrast, child flourishing and family resilience have emerged as key resources that enable adaptive coping, supportive relationships, and emotional strength in the face of trauma or chronic adversity.^13,14,15,16^ Flourishing reflects child and adolescent developmental competencies and adaptive functioning, whereas family resilience describes how families respond to stress, communicate effectively, and mobilize collective strengths when facing crisis or major family events.^13,14,15,16,17^ These constructs offer a strengths-based perspective that contrasts with traditional, deficit-focused risk models.^8,14^ Prior research demonstrates that child and adolescent flourishing is associated with greater emotional well-being, stronger school engagement, and fewer behavioral health problems in children and adolescents.^8,13,18,19^ Likewise, family resilience has been shown to be associated with buffering of stressors and improved psychosocial adjustment among children and adolescents with chronic illness.^20,21^

Guided by family resilience theory,^16,22^ we hypothesized that family resilience would modify the association between pediatric TBI and adverse mental and physical health outcomes. Although prior research has demonstrated that family environment and parenting style can moderate psychosocial outcomes after pediatric TBI,^15,23,24^ no study has quantitatively tested a validated family resilience construct as a moderator of mental and physical sequelae in a national pediatric TBI population. Using data from the 2022 to 2023 National Survey of Children’s Health (NSCH), we examined associations between medically diagnosed TBI and 4 key outcomes (anxiety, depression, frequent headaches, and chronic physical pain) and evaluated whether these associations varied by level of family resilience after accounting for demographics and socioeconomic status, ACEs, and child and adolescent flourishing index score. We hypothesized that higher family resilience would moderate the association between TBI and adverse outcomes.

## Methods

### Study Design and Participants

This cross-sectional study used data from the 2022 and 2023 NSCH, a nationally representative, cross-sectional survey conducted by the US Census Bureau and sponsored by the Health Resources and Services Administration Maternal and Child Health Bureau. The NSCH collects caregiver-reported data on health and well-being, health care access, family environments, and social determinants of health for children and adolescents aged 0 to 17 years across all 50 US states and the District of Columbia. This study used deidentified, publicly available data and was deemed exempt from review and consent by the Nationwide Children's Hospital Institutional Review Board under 45 CFR §46.104(d)(4). All analyses followed the Strengthening the Reporting of Observational Studies in Epidemiology (STROBE) reporting guideline for cross-sectional studies.

### Target Study Population

**Figure 1.**
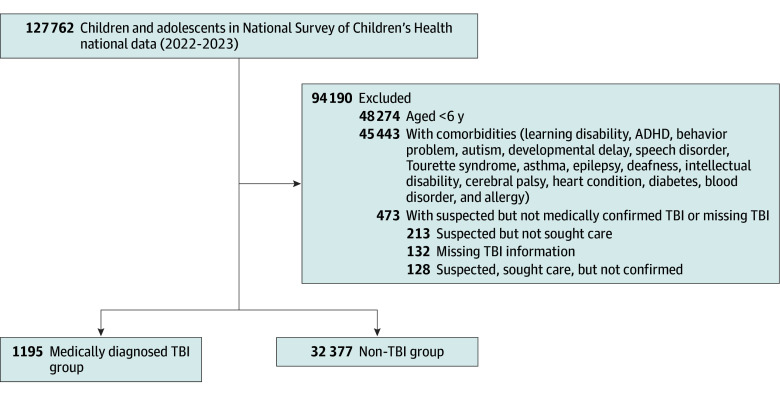
Study Flowchart ADHD indicates attention-deficit/hyperactivity disorder; TBI, traumatic brain injury.

To reduce confounding,^1,2,25^ we excluded children and adolescents with comorbid chronic physical and neurodevelopmental conditions, including allergies, asthma, attention-deficit disorder/attention-deficit hyperactivity disorder, autism spectrum disorder, behavioral problems (eg, conduct disorder and oppositional defiant disorder), blood disorders (eg, anemia and sickle cell disease), cerebral palsy, deafness or hearing loss, developmental delay, diabetes (type 1 or type 2), epilepsy or seizure disorders, heart conditions, intellectual disability, learning disabilities, speech disorders, or Tourette syndrome. A total of 45 443 children and adolescents, including 3006 individuals with TBI, were excluded (Figure 1).

We then derived a binary TBI variable. Children and adolescents were classified as having a medically diagnosed TBI if caregivers reported that the child or adolescent had a physician-confirmed diagnosis of TBI. Children and adolescents were classified as not having TBI if caregivers responded no to the concussion or brain injury question. We excluded 213 individuals whose caregivers suspected a concussion or brain injury but did not seek medical care, 128 individuals whose caregivers sought care but did not receive a physician-confirmed diagnosis of TBI, and 132 individuals with a missing brain injury answer. This more stringent classification ensured that the TBI group reflected clinically recognized cases while maintaining a clearly defined comparison group of peers without TBI.^1,23^ The final analytic sample comprised 1195 children and adolescents with medically diagnosed TBI and 32 377 children and adolescents without TBI (Figure 1).

### Study Covariate and Outcome Measurements

#### Main Covariates

All demographic, insurance, education, and income variables were reported by a parent or adult caregiver knowledgeable about the child or adolescent’s health as part of the NSCH. Child and adolescent age, sex, and race and ethnicity were identified by caregiver self-report in the household screener. Hispanic ethnicity was assessed separately from race, and respondents could select 1 or more racial categories. Race categories in the survey were American Indian or Alaska Native, Asian, Black, Native Hawaiian, and White; American Indian or Alaska Native, Asian, and Native Hawaiian were combined as other for this study due to small sample sizes. Ethnicity categories in the survey were Hispanic and non-Hispanic. Race and ethnicity were assessed because they are important social and structural determinants of health associated with differences in resource access, health care use, and health outcomes. Caregiver educational attainment was self-reported. Child and adolescent health insurance type reflected parent-reported current coverage. Household income was reported as total pretax family income in the prior calendar year. Federal poverty level status was derived from reported income and household size using federal poverty thresholds.

#### Outcomes

Core mental and physical health outcomes were constructed using validated NSCH items. Specifically, anxiety, depression, and frequent or severe headaches, including migraine headaches, were determined by a caregiver confirmative answer that a doctor or other clinician ever told the caregiver that the child or adolescent had the condition. If the parent or caregiver answered *yes*, then the parent or caregiver was asked to report whether the child or adolescent currently (when the survey was conducted) had the condition and, if so, the level of severity (mild, moderate, and severe) of the condition. Chronic physical pain was based on parent or caregiver reports of whether the child or adolescent had frequent or chronic difficulty due to physical pain over the past 12 months. All not applicable values in the raw data were treated as missing to ensure consistent handling across variables.

#### Child and Adolescent Flourishing and Family Resilience Index

Child and adolescent flourishing was measured using the Child Flourishing Index (CFI), a 3-item scale evaluating key developmental competencies, including whether the child or adolescent shows interest and curiosity in learning new things, works to finish tasks they start, and stays calm and in control when faced with a challenge. Each item was measured on a 4-point Likert scale (always to never). Consistent with prior NSCH studies,^8,17,19^ items were reverse coded and rescaled to 0 to 3, with higher scores reflecting greater flourishing. The 3 items were summed to create a composite CFI ranging from 0 to 9.^8,14,19^ We categorized CFI scores into low (range = 0-6), moderate (range = 7), or high (range = 8-9) levels. Categories were defined based on the empirical distribution of summed scores to balance group sizes for analysis.

Family resilience was assessed using a modified 6-item version of the Family Resilience Index (FRI) derived from the NSCH.^13,17^ The original FRI includes 4 caregiver-reported items designed to capture resilience-promoting behaviors and dynamics within families: (1) how often the family talks together about what to do, (2) works together to solve problems, (3) recognizes strengths to draw on, and (4) stays hopeful even in difficult times. As done by Bethell et al,^13,17^ to reflect broader family functioning, we added 2 items assessing caregiver-child connection (“can share ideas or talk about things that really matter with the child”) and parental coping (“handling the day-to-day demands of raising children”). All 6 items were rated on a 4-point Likert scale ranging from none of the time (0) to all of the time (3). Responses were summed to generate a composite score ranging from 0 to 18.^13,17^

Child and adolescent flourishing and family resilience measurements were based on child and adolescent development and family resilience theory and conceptual constructs.^16,26,27^ These measurements were validated and used by other investigators who used the NSCH.^8,13,17,19^ FRI scores were categorized into 3 levels of family resilience: low (0-14), moderate (15-17), and high (18).^13,17^ Cut points were selected to yield approximately balanced group sizes while maintaining adequate cell counts for analysis.

### Statistical Analysis

Statistical analysis followed the principles and steps recommended by the NSCH^28^ and used by other researchers.^8,13,17,19^ Full-year child and adolescent–level weights were adjusted for pooled data by dividing by 2, consistent with NSCH guidelines for multiyear estimates.^28^ The survey design included weights, stratification, and clustering variables.

First, descriptive analyses were performed to calculate weighted frequencies and proportions of children and adolescents with and without medically diagnosed TBI across sociodemographic variables (sex, age groups, race and ethnicity, family medical insurance type, highest education level of parents or caregivers, and household income relative to the federally defined poverty threshold [percentage FPL]). Then, weighted prevalence of medically diagnosed anxiety, depression, headaches, and caregiver-reported chronic physical pain was calculated separately for the 2 comparison groups (TBI vs non-TBI groups). We report the weighted prevalence by doctor or health professional–diagnosed condition, current status, and severity level of current anxiety, depression, and headaches. Bar graphs display the weighted prevalence (percentage) of children and adolescents aged 6 to 17 years with caregiver-reported current status of anxiety, depression, headache, or chronic physical pain, stratified by FRI level for TBI and non-TBI groups.

Adjusted prevalence ratios (aPRs) and 95% CIs were estimated using survey-weighted Poisson regression with Taylor series linearization to account for the NSCH’s complex sampling design,^1,29^ controlling for sex, age group, child or adolescent race or ethnicity, medical insurance type, highest education level of parents or caregiver, and percentage FPL level.

To evaluate associations between medically diagnosed TBI and parent or caregiver–reported current status of anxiety, depression, and headaches among children and adolescents, as well as associations between TBI and chronic physical pain, we fitted survey-weighted multivariable logistic regression models. Variables of main interest in our study were status of medically diagnosed TBI, number of ACEs, FRI level, and CFI level, while sex, age group, child or adolescent race and ethnicity, medical insurance type, highest education level of parents or caregiver, and household percentage FPL were considered as confounding factors. Results from multivariable logistic regression models provided evidence regarding whether there were associations between variables of interest (medically diagnosed TBI, ACEs, family resilience, and child or adolescent flourishing) and current status of anxiety, depression, headache, and chronic physical pain when other potential demographics, insurance type, parent or caregiver education, and household percentage FPL were controlled.

To test our study main hypothesis, we used survey-weighted multivariable logistic regression models with interaction terms to evaluate modification on the multiplicative (odds ratio [OR]) scale of FRI level on the association between medically diagnosed TBI and the previously listed 4 mental and physical outcomes (anxiety, depression, headache, and chronic physical pain).^8,15^ Logistic regression models included TBI status (yes and no) and FRI level (high, moderate, and low) as main effects. A TBI × FRI interaction term assessed effect modification on the multiplicative (OR) scale, testing whether associations between medically diagnosed TBI and caregiver-reported anxiety, depression, frequent headaches, and chronic physical pain varied by family resilience level. Controlled covariates included sex, age, race and ethnicity, medical insurance type, highest level of education among reported adults, household percentage FPL, ACEs, and CFI level.

Data were analyzed using R statistical software version 4.3.2 (R Project for Statistical Computing), with the survey package to account for the complex sampling design of the NSCH. Two-sided statistical significance was set at *P* < .05. Data analysis was done from June 1 to November 13, 2025.

## Results

### Sociodemographic Characteristics

A total of 33 572 participants, including 1195 individuals with medically diagnosed TBI (3.5%; 47.0% [95% CI, 41.2%-52.9%] female; 46.5% [95% CI, 41.0%-52.0%] aged 15-17 years; 3.4% [95% CI, 1.3%-5.5%] Black non-Hispanic, 21.4% [95% CI, 14.2%-28.6%] Hispanic, and 64.6% [95% CI, 58.0%-71.2%] White non-Hispanic) and 32 377 peers without TBI (96.4%; 54.0% [95% CI, 52.9%-55.1%] female; 51.7% [95% CI, 50.6%-52.8%] aged 6-11 years; 12.7% [95% CI, 11.8%-13.6%] Black non-Hispanic, 29.5% [95% CI, 28.4%-30.7%] Hispanic, and 44.7% [95% CI< 43.7%-45.8%] White non-Hispanic) were included in our study. Table 1 compares sociodemographic characteristics of groups with and without medically diagnosed TBI. For all demographic and family characteristics, children and adolescents with a history of a medically diagnosed TBI were significantly different from those without TBI. Given these imbalances, all multivariable models controlled for demographic and socioeconomic covariates, ACEs, flourishing, and family resilience, and findings were interpreted as associations.

**Table 1.** Sociodemographic Characteristics of Study Sample

Characteristic	Children and adolescents (No. = 33 572)						*P* value^a^
	Medically diagnosed TBI			No TBI			
	Sample No. (n = 1195)	Weighted No. (n = 657 290)	Weighted % (95% CI)	Sample No. (n = 32 377)	Weighted No. (n = 25 946 007)	Weighted % (95% CI)	
Sex							
Male	656	348 036	53.0 (47.1-58.8)	14 813	11 942 329	46.0 (44.9-47.1)	.02
Female	539	309 254	47.0 (41.2-52.9)	17 564	14 003 678	54.0 (52.9-55.1)	
Age group, y							
6-11	253	183 539	27.9 (21.3-34.5)	15 694	13 409 135	51.7 (50.6-52.8)	<.001
12-14	278	168 254	25.6 (21.1-30.1)	7719	6 658 933	25.7 (24.7-26.6)	
15-17	664	305 498	46.5 (41.0-52.0)	8964	5 877 940	22.7 (21.8-23.5)	
Race and ethnicity							
Black non-Hispanic	17	22 366	3.4 (1.3-5.5)	1990	3 290 460	12.7 (11.8-13.6)	<.001
Hispanic	120	140 841	21.4 (14.2-28.6)	5210	7 660 018	29.5 (28.4-30.7)	
White non-Hispanic	953	424 564	64.6 (58.0-71.2)	20 167	11 608 386	44.7 (43.7-45.8)	
Other non-Hispanic^b^	105	69 519	10.6 (7.9-13.3)	5010	3 387 143	13.1 (12.4-13.7)	
Medical insurance							
Public insurance only	133	108 699	16.5 (9.7-23.3)	6390	7 081 480	27.3 (26.3-28.3)	<.001
Private insurance only	972	490 832	74.7 (68.1-81.3)	22 453	14 977 052	57.7 (56.6-58.8)	
Other (private and public)	28	14 024	2.1 (1.0-3.2)	1085	1 064 198	4.1 (3.6-4.6)	
Uninsured	36	20 641	3.1 (1.6-4.7)	1712	2 075 725	8.0 (7.2-8.8)	
Missing value	26	23 093	3.5 (1.6-5.4)	737	747 553	2.9 (2.5-3.2)	
Highest level of education among reported adults							
<High school	17	40 972	6.2 (−0.9-13.3)	1148	2 868 986	11.1 (10.1-12.0)	.05
High school diploma	100	68 148	10.4 (7.5-13.2)	4441	4 998 772	19.3 (18.4-20.1)	
>High school	1078	548 170	83.4 (76.5-90.3)	26 788	18 078 249	69.7 (68.6-70.8)	
Family poverty ratio, % FPL^c^							
<100	61	65 323	9.9 (2.9-17.0)	3274	4 052 186	15.6 (14.7-16.5)	<.001
100-199	129	85 047	12.9 (9.6-16.3)	5411	5 510 271	21.2 (20.3-22.2)	
200-399	404	190 525	29.0 (24.8-33.2)	11 908	9 135 089	35.2 (34.2-36.2)	
≥400	601	316 396	48.1 (42.5-53.8)	11 784	7 248 461	27.9 (27.1-28.8)	

Abbreviations: FPL, federal poverty level; TBI, traumatic brain injury.

^a^ *P* values are derived from Rao-Scott adjusted χ^2^ tests, which test the independence between TBI status and each sociodemographic characteristic while properly accounting for survey design and weighting. Missing value amounts were not included in the calculation of *P* values.

^b^ Other non-Hispanic race includes American Indian or Alaska Native, Asian, and Native Hawaiian.

^c^ FPL was based on household size and total pretax family income reported for the prior calendar year. Family poverty ratio was calculated as the ratio of household income to the US Census Bureau federal poverty threshold for a family of the same size and composition in the survey year and categorized according to standard National Survey of Children’s Health groupings (eg, <100%, 100%-199%, 200%-399%, and ≥400% of FPL).

### Mental and Physical Health Sequelae

Children and adolescents with a history of medically diagnosed TBI had substantially higher rates of mental and physical health problems than their peers without TBI (Table 2). The weighted prevalence of current anxiety was 12.9% (95% CI, 9.3%-16.5%) among children and adolescents with TBI vs 4.7% (95% CI, 4.3%-5.0%) among those without TBI (aPR, 1.83 [95% CI, 1.38-2.43]). Current depression was also more common in the TBI group (6.7% [95% CI, 3.8%-9.6%]) than the non-TBI group (1.9% [95% CI, 1.6%-2.2%]; aPR, 2.13 [95% CI, 1.33-3.40]). A higher proportion of children and adolescents with TBI vs those without TBI had current frequent or severe headaches (10.8%, 95% CI, 3.5%-18.1% vs 1.2%, 95% CI, 1.0%-1.4%; aPR, 6.88 [95% CI, 2.73-17.34]) and chronic physical pain in the past 12 months (13.9%, 95% CI, 6.8%-20.9% vs 3.9% [95% CI, 3.4%-4.3%]; aPR, 3.38 [95% CI, 1.91-5.99]).

**Table 2.** Prevalence and aPR of Adverse Health Outcomes

Outcome	Children and adolescents (No. = 33 572)						aPR (95% CI)^a^
	Medically diagnosed TBI			No TBI			
	Sample No.	Weighted No.	Weighted % (95% CI)	Sample No.	Weighted No.	Weighted % (95% CI)	
**Anxiety**							
Doctor or health professional ever told							
Yes	186	92 725	14.6 (10.9-18.3)	2459	1 412 712	5.6 (5.2-6.0)	1.75 (1.36-2.25)
No	982	541 314	85.4 (81.7-89.1)	29 075	23 675 641	94.4 (94.0-94.7)	1 [Reference]
Current anxiety							
Yes	157	81 430	12.9 (9.3-16.5)	2101	1 174 119	4.7 (4.3-5.0)	1.83 (1.38-2.43)
No	1006	549 368	87.1 (83.5-90.7)	29 397	23 884 444	95.3 (95.0-95.7)	1 [Reference]
Current anxiety severity level							
Mild	82	48 616	59.7 (46.2-73.2)	1199	712 944	60.8 (57.3-64.3)	1 [Reference]
Moderate	65	26 354	32.4 (20.0-44.7)	809	418 406	35.7 (32.3-39.1)	0.95 (0.66-1.38)
Severe	10	6460	7.9 (1.8-14.0)	88	41 083	3.5 (2.3-4.7)	2.65 (1.27-5.54)
**Depression**							
Doctor or health professional ever told							
Yes	109	53 922	8.5 (5.5-11.5)	1068	645 965	2.6 (2.2-2.9)	2.13 (1.45-3.11)
No	1059	579 045	91.5 (88.5-94.5)	30 472	24 488 233	97.4 (97.1-97.8)	1 [Reference]
Current depression							
Yes	75	42 293	6.7 (3.8-9.6)	815	479 866	1.9 (1.6-2.2)	2.13 (1.33-3.40)
No	1092	590 619	93.3 (90.4-96.2)	30 713	24 645 927	98.1 (97.8-98.4)	1 [Reference]
Current depression severity level							
Mild	39	24 234	57.3 (36.6-77.9)	449	249 093	52.0 (43.7-60.2)	1 [Reference]
Moderate	29	12 430	29.4 (11.8-47.0)	303	198 387	41.3 (32.5-50.1)	0.89 (0.57-1.40)
Severe	7	5629	13.3 (2.0-24.7)	62	32 370	6.7 (4.0-9.5)	3.03 (1.34-6.83)
**Frequent or severe headaches**							
Doctor or health professional ever told							
Yes	103	90 032	14.2 (6.9-21.6)	701	460 344	1.8 (1.5-2.1)	6.14 (2.99-12.63)
No	1061	542 136	85.8 (78.4-93.1)	30 872	24 664 563	98.2 (97.9-98.5)	1 [Reference]
Current headaches							
Yes	75	68 260	10.8 (3.5-18.1)	491	297 352	1.2 (1.0-1.4)	6.88 (2.73-17.34)
No	1087	563 554	89.2 (81.9-96.5)	31 066	24 820 786	98.9 (98.6-99.0)	1 [Reference]
Current headache severity level							
Mild	32	21 213	31.1 (6.2-56.0)	273	170 024	57.7 (50.0-65.4)	1 [Reference]
Moderate	35	44 288	64.9 (37.4-92.5)	194	114 555	38.9 (31.3-46.5)	1.25 (0.86-1.80)
Severe	7	2689	3.9 (0.6-8.5)	19	9886	3.4 (0.1-6.6)	1.24 (0.30-5.13)
**Chronic physical pain in past 12 mo**							
Yes	135	87 716	13.9 (6.8-20.9)	1237	971 620	3.9 (3.4-4.3)	3.38 (1.91-5.99)
No	1031	544 796	86.1 (79.0-93.2)	30 293	24 115 987	96.1 (95.6-96.6)	1 [Reference]

Abbreviations: aPR, adjusted prevalence ratio; TBI, traumatic brain injury.

^a^ aPR and 95% CI were calculated using survey-weighted Poisson regression with standard errors, controlling for sex, age group, child or adolescent race and ethnicity, medical insurance type, highest education level of parents or caregivers, and household income level (percentage federal poverty level).

Children and adolescents with TBI were more likely to report moderate to severe symptoms of anxiety and depression than those without TBI. For example, the prevalence of severe anxiety among children and adolescents with TBI (7.9% [95% CI, 1.8%-14.0%]) was approximately double that of their peers without TBI (3.5% [95% CI, 2.3%-4.7%]; aPR, 2.65 [95% CI, 1.27-5.54); this was also true for severe depression (13.3% [95% CI, 2.0%-24.7%] vs 6.7% [95% CI, 4.0%-9.5%]; aPR, 3.03 [95% CI, 1.34-6.83]).

**Figure 2.**
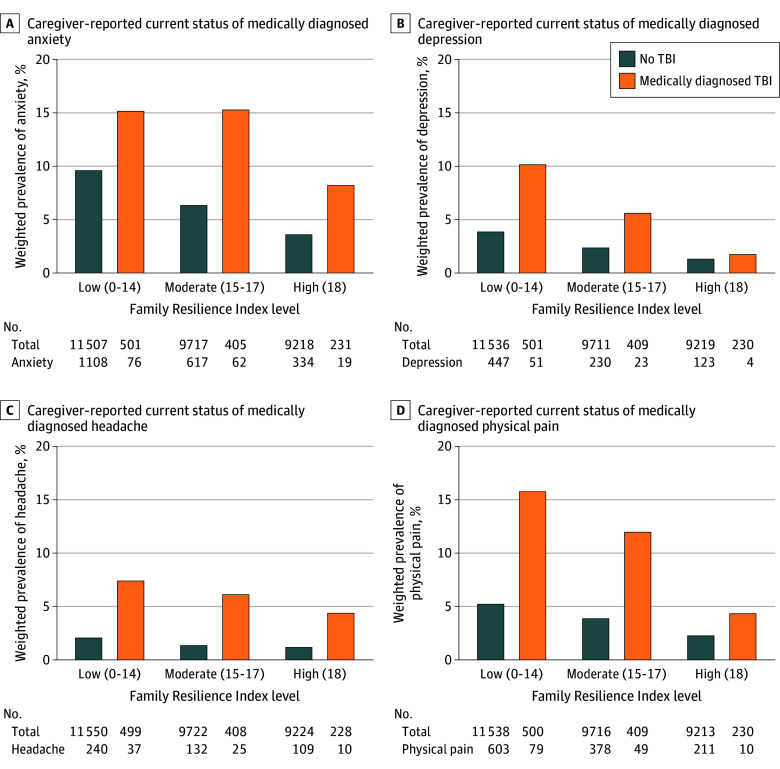
Bar Graphs of Weighted Prevalence of Mental and Physical Health Conditions TBI indicates traumatic brain injury.

As illustrated in Figure 2, mental and physical health problems decreased progressively with increasing levels of family resilience. Among children and adolescents with TBI, the prevalence of anxiety, depression, headache, and chronic pain was highest in the low-FRI group and lowest in the high-FRI group, demonstrating a graded pattern of association. This gradient was evident for mental and physical outcomes, whereas children and adolescents without TBI showed smaller differences across FRI levels.

### ACEs, Child Flourishing, and Family Resilience

In multivariable logistic regression models (Table 3), children and adolescents with medically diagnosed TBI had significantly higher adjusted odds of anxiety (adjusted OR [aOR], 1.87 [95% CI, 1.31-2.67]), depression (aOR, 1.98 [95% CI, 1.13-3.47]), frequent headaches (aOR, 7.76 [95% CI, 2.71-22.20]), and chronic physical pain (aOR, 3.99 [95% CI, 1.81-8.77]) compared with children and adolescents without TBI. Greater exposure to ACEs was associated with all outcomes, with particularly high point estimates for depression (aOR for ≥3 vs 0 ACES, 7.89 [95% CI, 5.18-12.02]) and anxiety (aOR for ≥3 vs 0 ACES, 3.76 [95% CI, 2.88-4.91]).

**Table 3.** Multivariable Logistic Regression Analysis of Association of Exposures With Current Status of Mental and Physical Health

Health Outcome	OR (95% CI)	*P* value	aOR (95% CI)^a^	*P* value
**Anxiety**				
TBI status				
No TBI	1 [Reference]	NA	1 [Reference]	NA
Medically diagnosed TBI	3.11 (2.21-4.39)	<.001	1.87 (1.31-2.67)	<.001
Reported ACEs, No.^b^				
0	1 [Reference]	NA	1 [Reference]	NA
Low (1)	1.53 (1.26-1.86)	<.001	1.51 (1.23-1.86)	<.001
Moderate (2)	2.26 (1.75-2.92)	<.001	2.11 (1.61-2.76)	<.001
High (≥3)	3.76 (2.97-4.76)	<.001	3.76 (2.88-4.91)	<.001
Family Resilience Index score^c^				
High (18)	1 [Reference]	NA	1 [Reference]	NA
Moderate (15-17)	2.00 (1.60-2.49)	<.001	1.35 (1.07-1.69)	.01
Low (0-14)	2.97 (2.41-3.65)	<.001	1.49 (1.19-1.88)	<.001
Child Flourishing Index score^d^				
High (8-9)	1 [Reference]	NA	1 [Reference]	NA
Moderate (7)	1.99 (1.54-2.55)	<.001	1.73 (1.33-2.24)	<.001
Low (0-6)	3.92 (3.17-4.83)	<.001	2.99 (2.37-3.77)	<.001
**Depression**				
TBI status				
No TBI	1 [Reference]	NA	1 [Reference]	NA
Medically diagnosed TBI	3.77 (2.26-6.27)	<.001	1.98 (1.13-3.47)	.02
Reported ACEs, No.^b^				
0	1 [Reference]	NA	1 [Reference]	NA
Low (1)	2.86 (1.74-4.69)	<.001	2.24 (1.51-3.33)	<.001
Moderate (2)	4.94 (3.42-7.13)	<.001	3.42(2.33-5.01)	<.001
High (≥3)	10.75 (7.42-15.56)	<.001	7.89(5.18-12.02)	<.001
Family Resilience Index score^c^				
High (18)	1 [Reference]	NA	1 [Reference]	NA
Moderate (15-17)	1.76 (1.12-2.79)	.02	1.14 (0.75-1.71)	.54
Low (0-14)	3.70 (2.31-5.93)	<.001	1.51 (0.99-2.29)	.05
Child Flourishing Index score^d^				
High (8-9)	1 [Reference]	NA	1 [Reference]	NA
Moderate (7)	1.34 (0.80-2.25)	.27	1.30 (0.79-2.14)	.30
Low (0-6)	4.27 (2.64-6.93)	<.001	3.17 (2.00-5.04)	<.001
**Headaches**				
TBI status				
No TBI	1 [Reference]	NA	1 [Reference]	NA
Medically diagnosed TBI	10.23 (4.63-22.60)	<.001	7.76 (2.71-22.20)	<.001
Reported ACEs, No.^b^				
0	1 [Reference]	NA	1 [Reference]	NA
Low (1)	1.60 (1.03-2.49)	.04	1.37 (0.87-2.18)	.18
Moderate (2)	1.18 (0.72-1.95)	.51	0.91 (0.52-1.60)	.75
High (≥3)	2.14 (1.19-3.88)	.01	1.46 (0.80-2.66)	.21
Family Resilience Index score^c^				
High (18)	1 [Reference]	NA	1 [Reference]	NA
Moderate (15-17)	1.71 (0.98-2.98)	.06	1.54 (0.83-2.85)	.17
Low (0-14)	1.95 (1.33-2.85)	<.001	1.64 (1.01-2.67)	.047
Child Flourishing Index score^d^				
High (8-9)	1 [Reference]	NA	1 [Reference]	NA
Moderate (7)	1.34 (0.75-2.38)	.33	1.28 (0.74-2.22)	.38
Low (0-6)	1.24 (0.72-2.14)	.44	1.00 (0.59-1.68)	1.00
**Chronic physical pain**				
TBI status				
No TBI	1 [Reference]	NA	1 [Reference]	NA
Medically diagnosed TBI	4.32 (2.31-8.09)	<.001	3.99 (1.81-8.77)	<.001
Reported ACEs, No.^b^				
0	1 [Reference]	NA	1 [Reference]	NA
Low (1)	1.60 (1.15-2.23)	.01	1.19 (0.87-1.62)	.29
Moderate (2)	1.99 (1.37-2.90)	<.001	1.25 (0.85-1.84)	.25
High (≥3)	3.72 (2.66-5.21)	<.001	2.30 (1.60-3.31)	<.001
Family Resilience Index score^c^				
High (18)	1 [Reference]	NA	1 [Reference]	NA
Moderate (15-17)	2.18 (1.57-3.04)	<.001	1.83 (1.25-2.68)	.002
Low (0-14)	3.16 (2.37-4.22)	<.001	2.08 (1.48-2.92)	<.001
Child Flourishing Index score^d^				
High (8-9)	1 [Reference]	NA	1 [Reference]	NA
Moderate (7)	1.86 (1.26-2.75)	.002	1.75 (1.17-2.61)	.01
Low (0-6)	2.63 (1.82-3.81)	<.001	1.95 (1.36-2.81)	<.001

Abbreviations: ACE, adverse childhood experience; aOR, adjusted odds ratio; NA, not applicable; OR, odds ratio; TBI, traumatic brain injury.

^a^ Multivariable models adjusted for sex, age, race and ethnicity, medical insurance type, highest level of education among reported adults, and family poverty ratio level.

^b^ ACEs were self-reported by the caregiver in the National Survey of Children’s Health: difficulty covering basic needs, such as food or housing (ACE1; 1 = never to 4 = very often); parental divorce or separation (ACE3); parental death (ACE4); parental time in jail or prison (ACE5); witnessing domestic violence in the home (ACE6); experiencing or witnessing neighborhood violence (ACE7); living with someone who was mentally ill, suicidal, or severely depressed (ACE8); living with someone who had problems with alcohol or drugs (ACE9); being treated or judged unfairly because of race or ethnicity (ACE10); and being treated or judged unfairly because of a health condition or disability (ACE11). ACE3 through ACE11 were coded as 1 = yes and 2 = no.

^c^ Family Resilience Index score was calculated using the National Survey of Children’s Health items, including how often families stay hopeful facing problems, talk together about what to do, work together to solve problems, and draw on strengths, each coded 1 = all of the time, 2 = most of the time, 3 = some of the time, and 4 = none of the time, and how well the caregiver and child can share ideas or talk about things that really matter (1 = very well to 4 = not well at all) and how well the caregiver thinks they are handling the day-to-day demands of raising children (1 = very well to 4 = not well at all).

^d^ Child Flourishing Index score was calculated using the National Survey of Children’s Health items, including caregiver-reported answers concerning whether the child shows interest and curiosity in learning new things, works to finish tasks they start, and stays calm and in control when faced with a challenge (each reverse coded as 0 = never, 1 = sometimes, 2 = usually, and 3 = always).

Low levels of child and adolescent flourishing were also associated with increased odds of anxiety (aOR, 2.99 [95% CI, 2.37-3.77]), depression (aOR, 3.17 [95% CI, 2.00-5.04]), and chronic pain (aOR, 1.95 [95% CI, 1.36-2.81]) compared with high flourishing. Similarly, children and adolescents from families with low resilience had increased odds of anxiety (aOR, 1.49 [95% CI, 1.19-1.88]), headaches (aOR, 1.64 [95% CI, 1.01-2.67]), and physical pain (aOR, 2.08 [95% CI, 1.48-2.92]) than those from families with high resilience.

### Interaction Between TBI and Family Resilience

As shown in the eTable in Supplement 1, family resilience modified the association between TBI and several health outcomes. Among children and adolescents without TBI, lower family resilience was associated with increased odds of anxiety (aOR, 1.36 [95% CI, 1.07-1.72]; *P* = .01), headaches (aOR, 1.79 [95% CI, 1.07-2.99]; *P* = .03), and chronic pain (aOR, 2.08 [95% CI, 1.47-2.94]; *P* < .001). In contrast, the association between TBI and depression varied by level of family resilience, with markedly higher odds at moderate (aOR, 5.64 [95% CI, 1.13-28.20]; *P* = .04) and low (aOR, 6.41 [95% CI, 1.24-33.20]; *P* = .03) resilience levels compared with the reference group (no TBI × high resilience). There were no interactions between TBI and family resilience for anxiety, headaches, or chronic physical pain, suggesting that family resilience primarily moderated the association between TBI and depressive outcomes.

## Discussion

This cross-sectional study used adult caregiver–reported data for 33 572 children and adolescents aged 6 to 17 years in the 2022 to 2023 NSCH to investigate the association between medically diagnosed TBI and mental and physical health outcomes. Our findings revealed that children and adolescents with TBI experienced significantly elevated rates of anxiety and depression and were markedly more likely than their peers without TBI to report severe current symptoms of these conditions. These findings, in line with prior studies,^2,23,30^ underscore the observed association of TBI with substantial increases in risks of adverse mental health indicators. Because we restricted TBI classification to caregiver-reported, physician-confirmed diagnoses and excluded suspected or unconfirmed cases, our findings likely reflected clinically recognized pediatric TBI and may represent conservative estimates relative to the broader spectrum of mild or unconfirmed TBI.

Additionally, our results showed that children and adolescents with TBI had increased odds of frequent headache and persistent chronic physical pain. These findings are consistent with previous research suggesting that chronic pain after TBI is multifactorial and involves neuroinflammatory processes, excitotoxicity, and altered pain-modulation pathways.^31,32,33,34,35^ These mechanisms may contribute not only to chronic headaches but also to chronic physical pain.^35^ Given the high prevalence and complexity of chronic headache and physical pain after TBI, early identification and multidisciplinary treatment approaches are essential to mitigate long-term disability and improve quality of life.^34,36,37^

Our interaction analysis of TBI × FRI level from survey-weighted multivariable logistic regression models found that children and adolescents with TBI who had low and moderate family resilience had a significantly increased risk of depression compared with the no TBI × high resilience group. Our findings contribute to a growing body of literature emphasizing the importance of family interventions, specifically family resilience, in mitigating adverse outcomes associated with TBI.^22,38^ Prior research showed that children and adolescents exposed to adversity, including TBI, had increased odds of emotional and behavioral challenges^24,39^; however, those embedded in a resilient family environment and who demonstrated flourishing traits, such as emotional regulation, curiosity, and persistence, tended to experience more favorable outcomes.^11,12,13^ Our results, in line with family resilience theory,^16,26^ suggest that family resilience was associated with decreased odds of adverse mental and physical outcomes, offering a critical pathway for early intervention and family support during recovery and rehabilitation after pediatric TBI.^24,38,40^ Unlike nonmodifiable factors, such as age, sex, intellectual abilities and education, and preinjury psychiatric history, family resilience is considered a key modifiable factor, alongside socioeconomic status, nutrition, and exercise, that may be associated with improved outcomes in multiple domains of functioning (eg, cognition, emotion regulation, health and wellness, and behavior) after a TBI.^38^ Studies have shown a small to medium positive effectiveness of family-oriented interventions after TBI for child and parental outcomes.^24,41,42,43,44^ Our findings of multiplicative-scale association modification provided new evidence for tailoring prevention efforts to subgroups most at risk, specifically, children and adolescents with a history of TBI who also report low levels of flourishing or family resilience.^4,24,38^

### Limitations

This study has limitations. First, the timing of the reported TBI relative to the interview and outcome ascertainment was unknown; therefore, temporal ordering could not be established in the study and reverse causation may be plausible. Second, its cross-sectional design precludes conclusions about temporal or causal relationships between TBI and subsequent mental and physical health outcomes. In addition, interaction analyses were conducted on the multiplicative (OR) scale and reflect associational effect modification rather than additive interaction or causal synergism. Longitudinal studies are needed to clarify the directionality of these associations. Third, all measures, including TBI history, mental and physical health outcomes, and family resilience and flourishing, were based on caregiver report, which may introduce recall and social desirability bias. Fourth, although analyses adjusted for a range of demographic and socioeconomic covariates, residual confounding from unmeasured variables, such as parental mental health, family coping style, or access to rehabilitation services, cannot be excluded. Fifth, findings are generalizable only to the US household–based population of children and adolescents aged 6 to 17 years and may not extend to younger children or institutionalized youths.

## Conclusions

This cross-sectional study found that family resilience was associated with decreased odds of depression after pediatric TBI. Clinically, these results underscore the value of integrating strength-based, family resilience–enhancing approaches into pediatric TBI care to promote holistic recovery and well-being. Future research should focus on developing and rigorously testing such interventions, alongside dissemination and implementation studies to identify and overcome barriers to their adoption, adaptation, and long-term sustainability.

## Notes

Supplement 1. **eTable.** Interaction of TBI Status and Family Resilience Level in Multivariable Logistic Regression Models of Current Status of Anxiety, Depression, Headaches, and Chronic Physical Pain among Children and Adolescents Aged 6-17 Years, NSCH 2022-2023

Supplement 2.
Data Sharing Statement
