# Supplementary material for: Family Resilience and Mental and Physical Health Sequelae of Pediatric TBI in Youths
Source: JAMA Netw Open. 2026 Apr 13;9(4):e269222. doi: 10.1001/jamanetworkopen.2026.9222 (PMC13077510; doi:10.1001/jamanetworkopen.2026.9222)
Supplement: Supplement 1. — eTable. Interaction of TBI Status and Family Resilience Level in Multivariable Logistic Regression Models of Current Status of Anxiety, Depression, Headaches, and Chronic Physical Pain among Children and Adolescents Aged 6-17 Years, NSCH 2022-2023 [file jamanetwopen-e269222-s001.pdf]

Supplemental Online Content

Zhou Z, Sullivan L, Qian R, et al. Family resilience and mental and physical health sequelae of pediatric TBI in youths. *JAMA Netw Open*. 2026;9(4):e269222. doi:10.1001/jamanetworkopen.2026.9222

eTable. Interaction of TBI Status and Family Resilience Level in Multivariable Logistic Regression Models of Current Status of Anxiety, Depression, Headaches, and Chronic Physical Pain among Children and Adolescents Aged 6-17 Years, NSCH 2022-2023

This supplemental material has been provided by the authors to give readers additional information about their work.

© 2026 Zhou Z et al. *JAMA Network Open*.

eTable. Interaction of TBI Status and Family Resilience Level in Multivariable Logistic Regression Models of Current Status of Anxiety, Depression, Headaches, and Chronic Physical Pain among Children and Adolescents Aged 6-17 Years, NSCH 2022-2023

Interaction Term§	Anxiety		Depression		Headaches		Chronic Physical Pain	
	Adj OR¥ (95% CI)	*P*-value	Adj OR¥ (95% CI)	*P*-value	Adj OR¥ (95% CI)	*P*-value	Adj OR¥ (95% CI)	*P*-value
No TBI × FRI-High	ref		ref		ref		ref	
No TBI × FRI-Moderate	1.36 (1.07-1.72)	0.01	1.08 (0.71-1.65)	0.72	1.24 (0.77-2.00)	0.37	1.66 (1.17-2.34)	0.00
No TBI × FRI-Low	1.53 (1.21-1.93)	<0.001	1.42 (0.94-2.16)	0.10	1.79 (1.07-2.99)	0.03	2.08 (1.47-2.94)	<0.001
TBI × FRI-High	2.56 (1.34-4.87)	0.00	0.36 (0.09-1.49)	0.16	6.12 (2.46-15.20)	<0.001	1.88 (0.76-4.63)	0.17
TBI × FRI-Moderate	0.82 (0.37-1.83)	0.62	5.64 (1.13-28.20)	0.04	2.74 (0.63-11.97)	0.18	3.86 (0.89-16.84)	0.07
TBI × FRI-Low	0.62 (0.26-1.48)	0.29	6.41 (1.24-33.20)	0.03	0.56 (0.20-1.62)	0.29	1.36 (0.51-3.67)	0.54

Abbreviations: NSCH, National Survey of Children's Health; TBI, Traumatic brain injury; FRI, Family resilience level; Adj OR, adjusted odds ratio; CI, Confidence interval.

¥ Survey-weighted multivariable logistic regression models were conducted separately for the caregiver reported current status of anxiety, depression, headaches, and chronic physical pain. Models were adjusted for sex, age, race/ethnicity, medical insurance type, highest level of education among reported adults, and family poverty ratio level (%FPL), number of adverse childhood experiences (ACEs), and Child Flourishing Index (CFI).

In these logistic regression models, traumatic brain injury (TBI) status (yes/no) and Family Resilience Index (FRI) level (high, moderate, low) were included as main effect terms.

§ The interaction term (TBI × FRI) represents the moderating effect of family resilience on the association between medically diagnosed TBI and caregiver-reported current status of anxiety, depression, frequent headaches, and chronic physical pain.

© 2026 Zhou Z et al. *JAMA Network Open*.
