# Supplementary material for: Family Resilience and Mental and Physical Health Sequelae of Pediatric TBI in Youths
Source: JAMA Netw Open. 2026 Apr 13;9(4):e269222. doi: 10.1001/jamanetworkopen.2026.9222 (PMC13077510; doi:10.1001/jamanetworkopen.2026.9222)
Supplement: Supplement 2. — Data Sharing Statement [file jamanetwopen-e269222-s002.pdf]

Data Sharing Statement

Zhou. Family Resilience and Mental and Physical Health Sequelae of Pediatric TBI in Youths.
JAMA Netw Open. Published online April 13, 2026. doi:10.1001/jamanetworkopen.2026.9222

Data
Data available: Yes
Data types: Deidentified participant data, Data dictionary
How to access data: The NSCH Datasets and data dictionary are already publicly available at the www.childhealthdata.org.
When available: With publication

Supporting Documents
Document types: None

Additional Information
Who can access the data: Anyone requesting the data.
Types of analyses: For any research purpose.
Mechanisms of data availability: The PI will share information about the datasets and data dictionary that are available in the public domain.
